# Supplementary material for: Predictive validity of the quick Sequential Organ Failure Assessment (qSOFA) score for the mortality in patients with sepsis in Vietnamese intensive care units
Source: PLoS One. 2022 Oct 14;17(10):e0275739. doi: 10.1371/journal.pone.0275739 (PMC9565713; doi:10.1371/journal.pone.0275739)
Supplement: S2 File — (PDF) [file pone.0275739.s002.pdf]

SUPPLEMENTARY RESULTS

Figure S1. Flowchart of the study design, patient enrollment and follow up

Figure S2. The area under the ROC curves of the qSOFA score for predicting the ICU mortality in patients with sepsis in Vietnamese ICUs

Figure S3. Comparing the areas under the ROC curves of the qSOFA and the qSOFA-65 scores for predicting hospital mortality

Figure S4. Comparing the areas under the ROC curves of the qSOFA and the qSOFA-65 scores for predicting ICU mortality

Table S1. Clinical, laboratory and microbiology characteristics and severity of illness upon intensive care unit admission according to hospital survivability of patients with sepsis

Table S2. Life-sustaining treatments during intensive care unit stay and outcomes according to hospital survivability of patients with sepsis

Table S3. Clinical, laboratory and microbiology characteristics and severity of illness upon intensive care unit (ICU) admission according to ICU survivability of patients with sepsis

Table S4. Life-sustaining treatments during intensive care unit (ICU) stay and outcomes according to ICU survivability of patients with sepsis

Table S5. Factors associated with hospital mortality in patients with sepsis upon intensive care unit admission: univariable regression analyses

Table S6. Factors associated with intensive care unit (ICU) mortality in patients with sepsis upon ICU admission: univariable regression analyses

Table S7. Factors (including the qSOFA score of 3) associated with hospital mortality in patients with sepsis upon intensive care unit admission: multivariable logistic regression analyses (backward elimination)

Table S8. Factors (including the qSOFA score of 3) associated with intensive care unit (ICU) mortality in patients with sepsis upon ICU admission: multivariable logistic regression analyses (backward elimination)

Table S9. Factors (including the qSOFA score of 2 to 3) associated with hospital mortality in patients with sepsis upon intensive care unit admission: multivariable logistic regression analyses (backward elimination)

Table S10. Factors (including the qSOFA score of 2 to 3) associated with intensive care unit (ICU) mortality in patients with sepsis upon ICU admission: multivariable logistic regression analyses (backward elimination)

Table S11. Breakdown of missing data

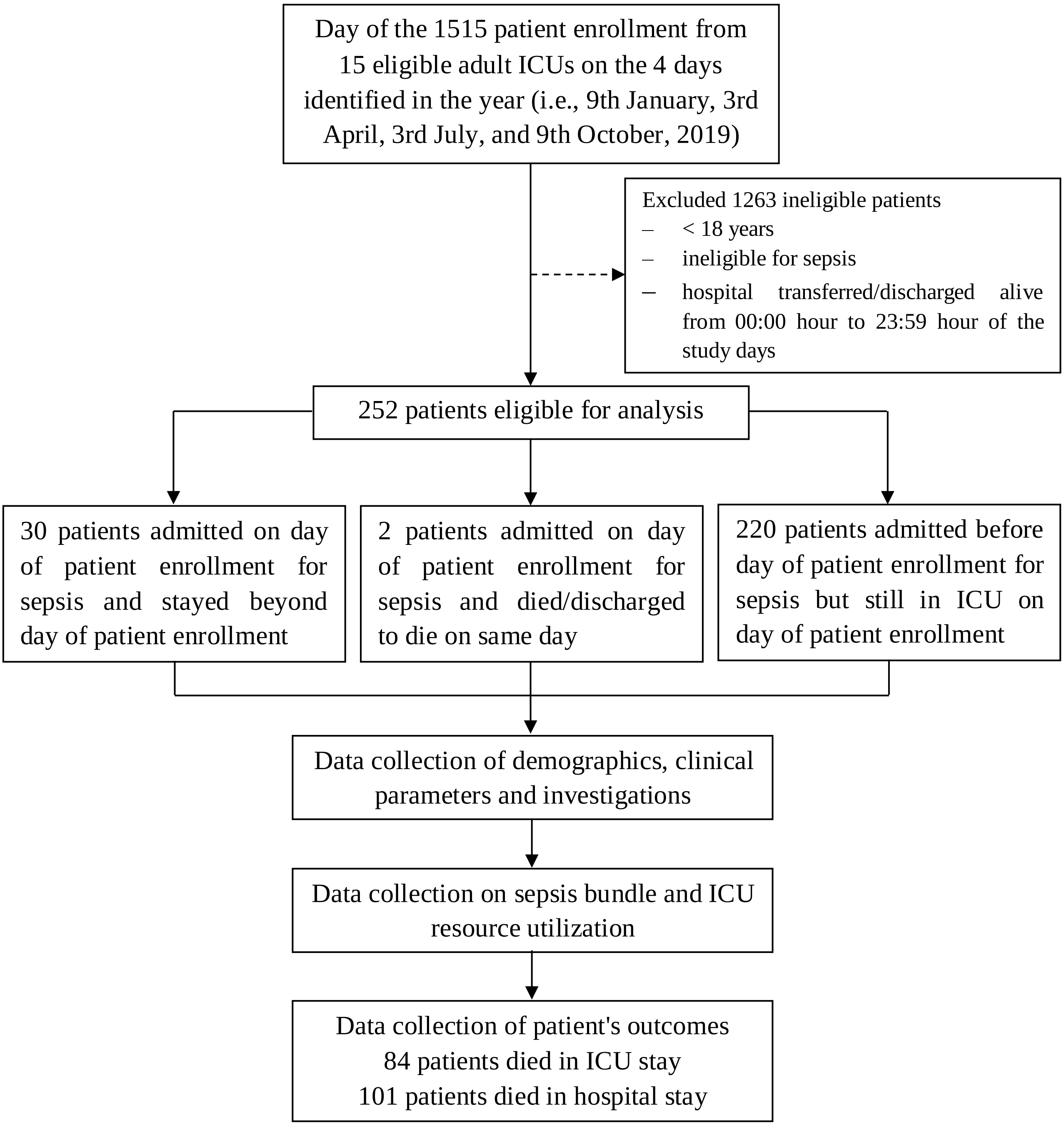

Figure S1. Flowchart of the study design, patient enrollment and follow up. Abbreviations: **ICU**, intensive care unit; **“discharged to die”**, defined as the patients were in grave condition or dying and were classified with a death in the ICU at the time of discharge.

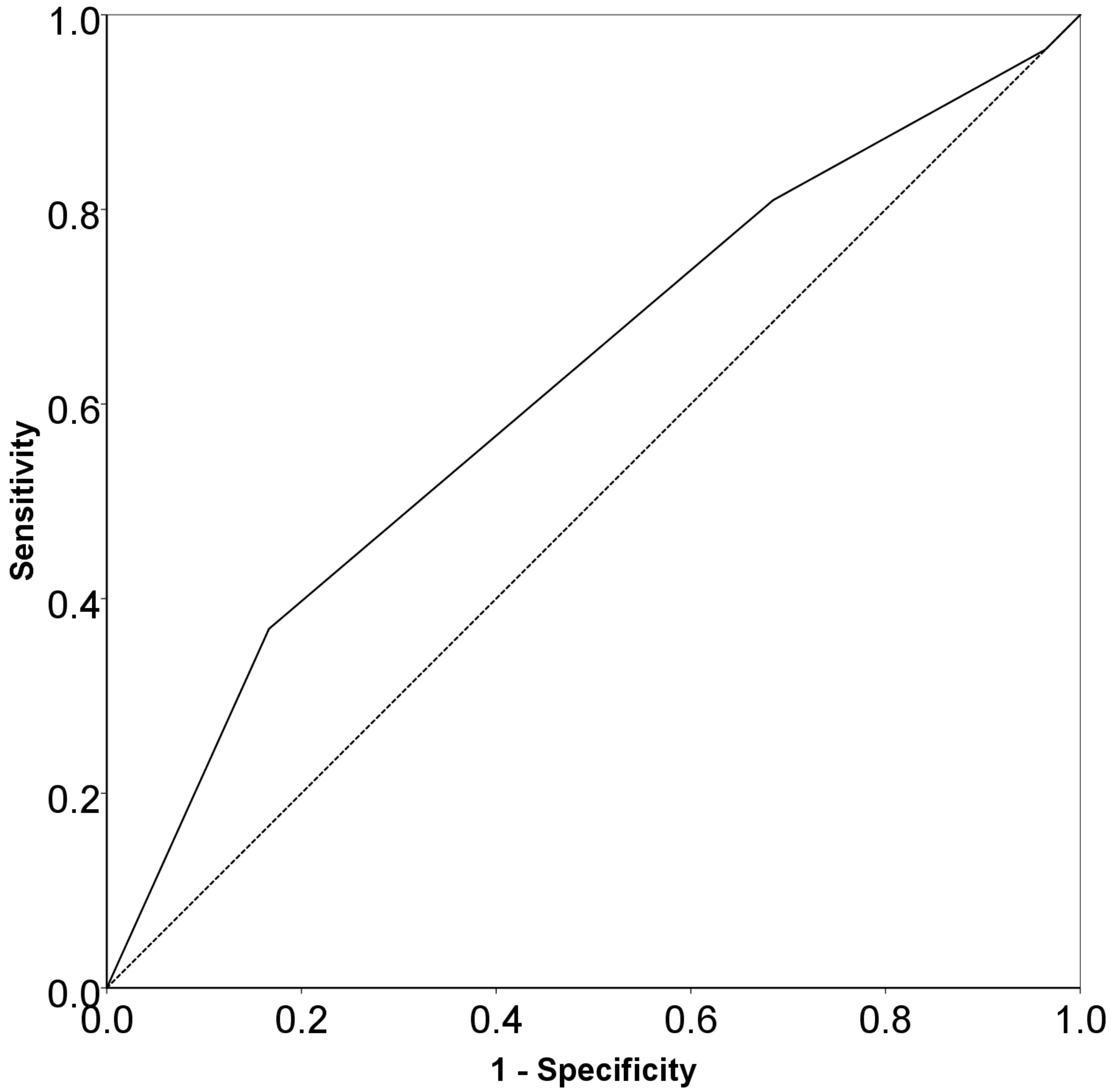

Figure S2. The area under the ROC curves of the qSOFA score (AUROC: 0.619 [95% CI: 0.544-0.694]; cut-off value: ≥2.5; sensitivity: 36.9%; specificity: 83.3%; P_{AUROC}=0.002) for predicting the ICU mortality in patients with sepsis in Vietnamese ICUs (Abbreviations: **AUROC**, area under the receiver operating characteristic curve; **CI**, confidence interval; **ICU**, intensive care unit; **qSOFA**, Quick Sequential Organ Failure Assessment; **ROC**, receiver operating characteristic; **SOFA**, Quick Sequential Organ Failure Assessment).

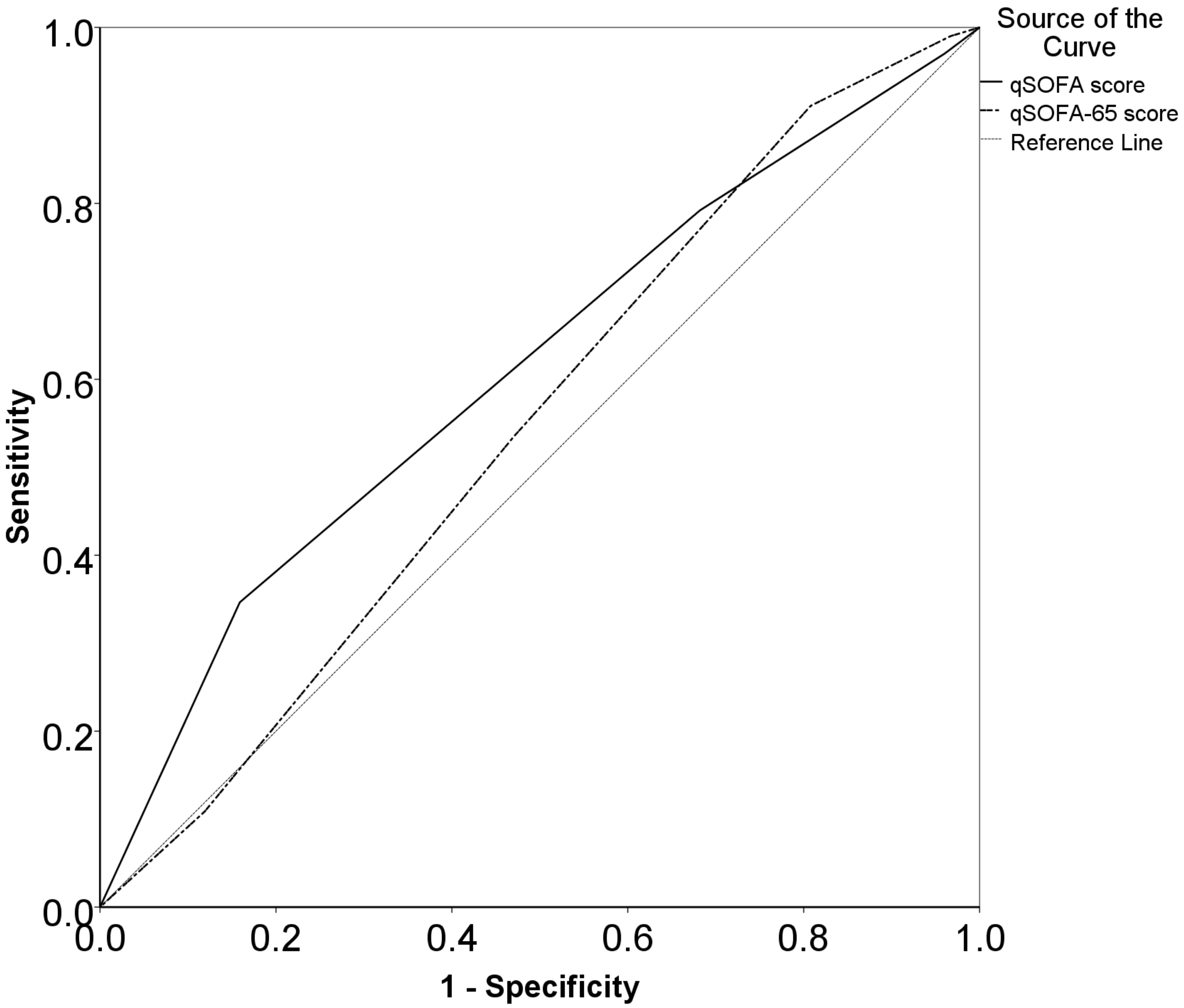

Figure S3. Comparing the areas under the ROC curves of the qSOFA and the qSOFA-65 scores for predicting hospital mortality. Comparing the overall diagnostic performance of the qSOFA (AUROC: 0.610 [95% CI: 0.538-0.681]; cut-off value ≥2.5; sensitivity: 34.7%; specificity: 84.1%; P_{AUROC}=0.003) and the qSOFA-65 (AUROC: 0.548 [95% CI: 0.476-0.619]; cut-off value ≥2.5; sensitivity: 53.5%; specificity: 53.0%; P_{AUROC}=0.201) scores for predicting the hospital mortality in patients with sepsis in ICUs (Abbreviations: **AUROC**, areas under the receiver operating characteristic curve; **CI**, confidence interval; **qSOFA**, Quick Sequential Organ Failure Assessment; **ROC**, receiver operating characteristic; **qSOFA-65**, defined as including the age criterion ≥65 years to the qSOFA score).

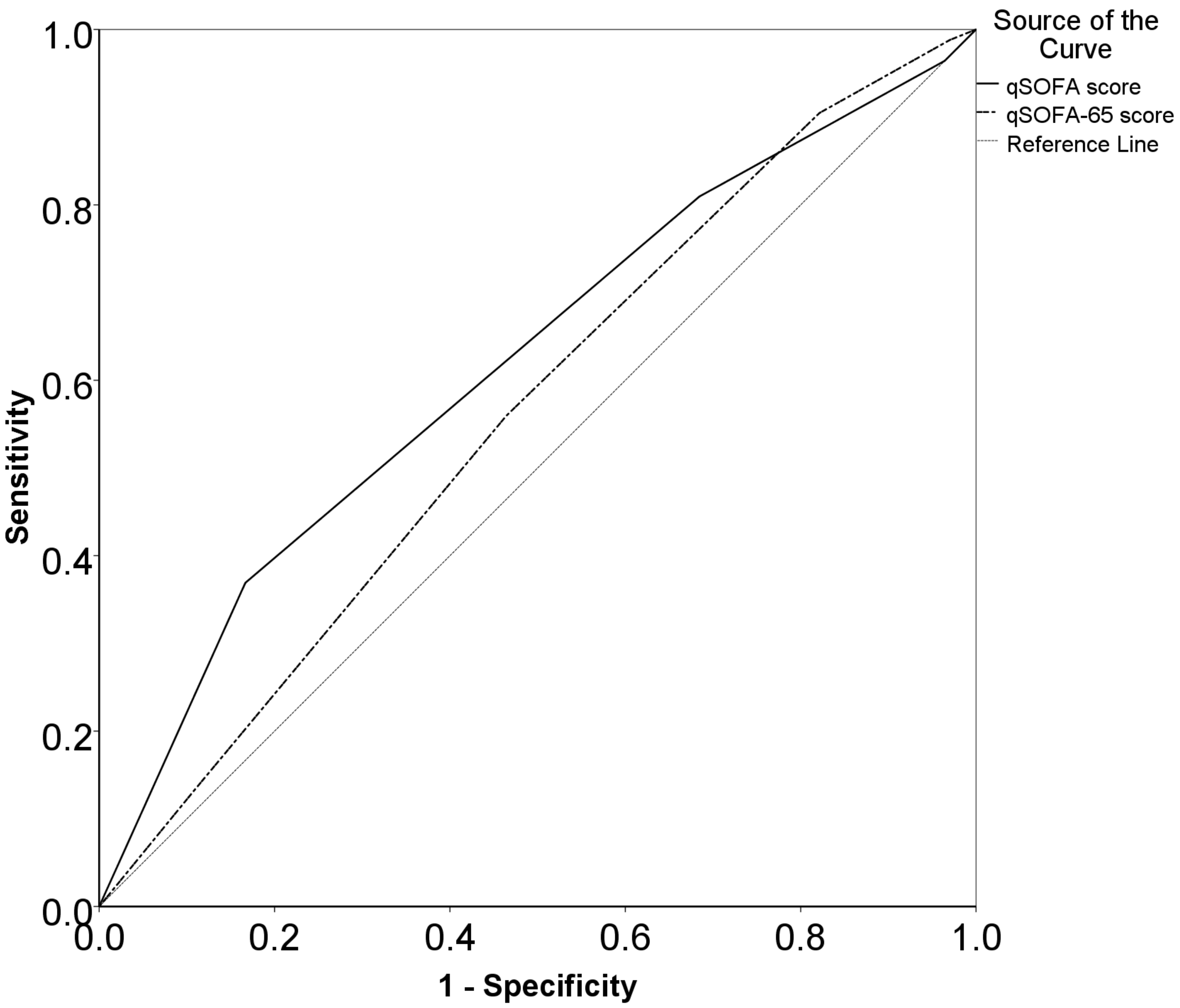

Figure S4. Comparing the areas under the ROC curves of the qSOFA and the qSOFA-65 scores for predicting ICU mortality. Comparing the overall diagnostic performance of the qSOFA (AUROC: 0.619 [95% CI: 0.544-0.694]; cut-off value ≥2.5; sensitivity: 36.9%; specificity: 83.3%; P_{AUROC}=0.002) and the qSOFA-65 (AUROC: 0.562 [95% CI: 0.488-0.636]; cut-off value ≥2.5; sensitivity: 56.0%; Specificity: 53.6%; P_{AUROC}=0.107) scores for predicting the ICU mortality in patients with sepsis in ICUs (Abbreviations: **AUROC**, areas under the receiver operating characteristic curve; **CI**, confidence interval; **ICU**, intensive care unit; **qSOFA**, Quick Sequential Organ Failure Assessment; **ROC**, receiver operating characteristic; **qSOFA-65**, defined as including the age criterion ≥65 years to the qSOFA score).

Table S1. Clinical, laboratory and microbiology characteristics and severity of illness upon intensive care unit admission according to hospital survivability of patients with sepsis

Variable	All cases n=252	Survived n=151	Died n=101	p-value[a]
Participating hospital, no. (%)				NA
115 People's	25 (9.9)	6 (4.0)	19 (18.8)	
Bach Mai[b]	26 (10.3)	14 (9.3)	12 (11.9)	
Bai Chay	14 (5.6)	10 (6.6)	4 (4.0)	
Can Tho[b]	7 (2.8)	1 (0.7)	6 (5.9)	
Cho Ray[b]	41 (16.3)	19 (12.6)	22 (21.8)	
Da Nang	12 (4.8)	6 (4.0)	6 (5.9)	
Dong Da	9 (3.6)	6 (4.0)	3 (3.0)	
Hanoi Medical University	12 (4.8)	6 (4.0)	6 (5.9)	
Hue[b]	39 (15.5)	26 (17.2)	13 (12.9)	
Saint Paul	9 (3.6)	9 (6.0)	0 (0.0)	
Thai Nguyen[b]	2 (0.8)	1 (0.7)	1 (1.0)	
Thanh Nhan	1 (0.4)	0 (0.0)	1 (1.0)	
Vietnam–Czechoslovakia Friendship	48 (19.0)	40 (26.5)	8 (7.9)	
Vinmec Times City International	7 (2.8)	7 (4.6)	0 (0.0)	
Age (year), median (Q1-Q3)	65 (52-77)	65 (53-76)	65 (52-78)	0.810**
Age (year), no. (%)				0.865*
< 20	3 (1.2)	2 (1.3)	1 (1.0)	
20 - 39	19 (7.5)	10 (6.6)	9 (8.9)	
40 - 59	74 (29.4)	43 (28.5)	31 (30.7)	
≥ 60	156 (61.9)	96 (63.6)	60 (59.4)	
Age (year), no. (%)				0.939
< 65	123 (48.8)	74 (49.0)	49 (48.5)	
≥ 65	129 (51.2)	77 (51.0)	52 (51.5)	
Sex (male), no. (%)	162 (64.3)	93 (61.6)	69 (68.3)	0.275
Documented comorbidities, no. (%)				
Cardiovascular disease	78 (31.0)	41 (27.2)	37 (36.6)	0.111

Chronic lung disease	30 (11.9)	18 (11.9)	12 (1.9)	0.992
Chronic neurological disease	36 (14.3)	28 (18.5)	8 (7.9)	0.018
Chronic kidney disease	23 (9.1)	14 (9.3)	9 (8.9)	0.922
Peptic ulcer disease	9 (3.6)	5 (3.3)	4 (4.0)	>0.999*
Chronic liver disease	27 (10.7)	14 (9.3)	13 (12.9)	0.365
Diabetes mellitus	67 (26.6)	40 (26.5)	27 (26.7)	0.966
HIV infection	0 (0.0)	0 (0.0)	0 (0.0)	NA
Connective tissue disease	3 (1.2)	2 (1.3)	1 (1.0)	>0.999*
Immunosuppression	10 (4.0)	7 (4.6)	3 (3.0)	0.744
Haematological malignancies	5 (2.0)	3 (2.0)	2 (2.0)	>0.999*
Solid malignant tumours	12 (4.8)	6 (4.0)	6 (5.9)	0.551*
**Vital signs** (on admission into ICU)				
GCS, median (Q1-Q3)	13 (9-15)	14 (10-15)	10 (8-14)	<0.001**
HR (beats per min), median (Q1-Q3)	110 (95-126)	110 (92-125)	110 (100-130)	0.083**
Temperature (^{o}C), mean (SD)	37.79 (1.01)	37.80 (1.08)	37.77 (0.91)	0.871**
MBP (mmHg), mean(SD)	75.82 (22.08)	79.75 (22.88)	69.93 (19.51)	0.002**
SBP (mmHg), mean (SD)	106.45 (29.96)	111.39 (29.44)	99.07 (29.35)	0.004**
RR (breaths per min), median (Q1-Q3)	25 (22-30)	25 (22-30)	25 (20-30)	0.693**
**Blood investigations**				
Total WBC (x10^9/L), mean (SD)	15.73 (9.20)	15.63 (8.67)	15.88 (9.98)	0.914**
PLT (x10^9/L), mean (SD)	185.98 (137.85)	200.71 (129.67)	163.95 (147.15)	0.002**
Hb (g/dL), mean (SD)	11.14 (2.59)	11.36 (2.68)	10.82 (2.44)	0.088**
Hct (%), mean (SD)	34.31 (7.75)	35.08 (7.92)	33.17 (7.38)	0.031**
K^+ (mmol/L), mean (SD)	3.89 (0.79)	3.90 (0.80)	3.87 (0.77)	0.865**
Na^+ (mmol/L), mean (SD)	136.05 (8.24)	135.62 (8.81)	136.69 (7.80)	0.068**
Creatinine (µmol/L), mean (SD)	187.85 (151.92)	186.15 (171.60)	190.38 (117.27)	0.030**

Bilirubin (µmol/l), mean (SD)	32.80 (61.49)	31.74 (72.67)	34.35 (40.09)	0.007**
pH, mean (SD)	7.37 (0.50)	7.41 (0.64)	7.32 (0.14)	0.004**
PaO_2 (mmHg), mean (SD)	116.17 (74.28)	110.23 (56.25)	124.73 (94.07)	0.665**
FiO_2, mean (SD)	0.50 (0.22)	0.44 (0.18)	0.58 (0.24)	<0.001**
PaO_2/FiO_2 ratio, mean (SD)	262.48 (149.58)	281.52 (149.39)	235.26 (146.32)	0.003**
**Severity of illness scores**				
qSOFA, median (Q1-Q3)	2 (1-2)	2 (1-2)	2 (2-3)	0.001**
qSOFA, no. (%)				0.006
0	9 (3.6)	6 (4.0)	3 (3.0)	
1	60 (23.8)	42 (27.8)	18 (17.8)	
2	124 (49.2)	79 (52.3)	45 (44.6)	
3	59 (23.4)	24 (15.9)	35 (34.7)	
qSOFA, no. (%)				0.055
0 - 1	69 (27.4)	48 (31.8)	21 (20.8)	
2 - 3	183 (72.6)	103 (68.2)	80 (79.2)	
qSOFA, no. (%)				0.001
0 - 2	193 (76.6)	127 (84.1)	66 (65.3)	
3	59 (23.4)	24 (15.9)	35 (34.7)	
SIRS criteria, median (Q1-Q3)	3 (2-4)	3 (2-4)	3 (2-4)	0.937**
SOFA score, median (Q1-Q3)	7 (5-10)	6 (4-9)	9 (6-12)	<0.001**
SOFA score, no. (%)				<0.001
< 8	140 (56.0)	104 (69.8)	36 (35.6)	
≥ 8	110 (44.0)	45 (30.2)	65 (64.4)	
APACHE II score, median (Q1-Q3)	18 (13-24)	15 (12-21)	22 (16-27)	<0.001**
APACHE II score, no. (%)				<0.001
< 19	129 (51.2)	97 (64.2)	32 (31.7)	
≥ 19	123 (48.8)	54 (35.8)	69 (68.3)	
Septic Shock, no. (%)	74 (29.4)	35 (23.2)	39 (38.6)	0.008
**Site of Infection**				

Respiratory, no. (%)	143 (56.7)	82 (54.3)	61 (60.4)	0.339
Urinary tract, no. (%)	37 (14.7)	30 (19.9)	7 (6.9)	0.004
Abdominal, no. (%)	61 (24.2)	34 (22.5)	27 (26.7)	0.444
Neurological, no. (%)	12 (4.8)	8 (5.3)	4 (4.0)	0.767*
Bones or joints, no. (%)	2 (0.8)	2 (1.3)	0 (0.0)	0.518*
Skin or cutaneous sites, no. (%)	19 (7.5)	7 (4.6)	12 (11.9)	0.033
Intravascular catheter, no. (%)	1 (0.4)	1 (0.7)	0 (0.0)	>0.999*
Infective endocarditis, no. (%)	1 (0.4)	0 (0.0)	1 (1.0)	0.401*
Primary bacteremia, no. (%)	7 (2.8)	5 (3.3)	2 (2.0)	0.705*
Systemic, no. (%)	6 (2.4)	4 (2.6)	2 (2.0)	>0.999*
**Microbiology**				
No pathogens detected, no. (%)	67 (26.6)	47 (31.1)	20 (19.8)	0.046
Gram negative bacteria, no. (%)	156 (61.9)	88 (58.3)	68 (67.3)	0.147
Klebsiella pneumonia	27 (10.7)	16 (10.6)	11 (10.9)	0.941
Acinetobacter baumannii	45 (17.9)	21 (13.9)	24 (23.8)	0.045
Escherichia coli	44 (17.5)	26 (17.2)	18 (17.8)	0.902
Pseudomonas aeruginosa	24 (9.5)	17 (11.3)	7 (6.9)	0.251
Stenotrophomonas maltophilia	2 (0.8)	0 (0.0)	2 (2.0)	0.160*
Proteus species	47 (18.7)	25 (16.6)	22 (21.8)	0.297
Enterobacter cloacae	3 (1.2)	3 (2.0)	0 (0.0)	0.277*
Bulkholderia pseudomallei	1 (0.4)	0 (0.0)	1 (1.0)	0.401*
Gram positive bacteria, no. (%)	34 (13.5)	22 (14.6)	12 (11.9)	0.540
Enterococcus	5 (2.0)	5 (3.3)	0 (0.0)	0.085*
MSSA	5 (2.0)	3 (2.0)	2 (2.0)	>0.999*
MRSA	10 (4.0)	6 (4.0)	4 (4.0)	>0.999*
Other Streptococcus species	12 (4.8)	6 (4.0)	6 (5.9)	0.551*
Streptococcus pneumonia	2 (0.8)	2 (1.3)	0 (0.0)	0.518*
Fungi, no. (%)	7 (2.8)	4 (2.6)	3 (3.0)	>0.999*
Candida species	7 (2.8)	4 (2.6)	3 (3.0)	>0.999*
Aspergillus species	0 (0.0)	0 (0.0)	0 (0.0)	NA
Viruses, no. (%)	2 (0.8)	0 (0.0)	2 (2.0)	0.160*
Influenza	1 (0.4)	0 (0.0)	1 (1.0)	0.401*

Dengue	1 (0.4)	0 (0.0)	1 (1.0)	0.401*
Other pathogens, no. (%)				
Anaerobes	0 (0.0)	0 (0.0)	0 (0.0)	NA
Mycobacterium tuberculosis	4 (1.6)	3 (2.0)	1 (1.0)	0.651*
Malaria	0 (0.0)	0 (0.0)	0 (0.0)	NA

[a]Comparison between the patients who survived and died using Chi-squared test; *Fisher's exact test; **Mann–Whitney U test.

[b]Central hospitals

Abbreviations

APACHE II score: Acute Physiology and Chronic Health Evaluation II score; **FiO_2**: fraction of inspired oxygen; **GCS**: Glasgow Coma Scale; **Hb**: hemoglobin; **Hct**: hematocrit; **HIV**: human immunodeficiency virus; **HR**: heart rate; **ICU**: intensive care unit; **MBP**: mean arterial blood pressure; **n**, total number of patient; **NA**, not available; **no.**, total number of patients recorded if a variable was given; **MRSA**: methicillin-resistant Staphylococcus aureus; **MSSA**: methicillin-susceptible Staphylococcus aureus; **PaO_2**: partial pressure of oxygen in the arterial blood; **PLT**: platelet; **Q**: quartile; **qSOFA score**: quick Sequential (Sepsis-Related) Organ Failure Assessment score; **RR**: respiration rate; **SBP**: systolic blood pressure; **SD**: standard deviation; **SIRS criteria**: Systemic Inflammatory Response Syndrome criteria; **SOFA score**: Sequential (Sepsis-Related) Organ Failure Assessment score; **WBC**: white blood cell.

Table S2. Life-sustaining treatments during intensive care unit stay and outcomes according to hospital survivability of patients with sepsis

Variable	All cases n=252	Survived n=151	Died n=101	p-value[a]
**Life-sustaining treatments during ICU stay**				
Respiratory support, no. (%) or median (Q1-Q3) days				
Mechanical ventilation[b]	173 (68.9)	82 (54.7)	91 (90.1)	<0.001
Duration of mechanical ventilation[b]	8 (4-15)	9 (4-15)	7(3-14)	0.153**
Non-invasive ventilation[b]	20 (8.0)	13 (8.7)	7 (6.9)	0.618
Duration of non-invasive ventilation[b]	2 (2-4)	2 (1-2)	5 (2-7)	0.004**
High-flow nasal oxygen[b]	38 (15.1)	29 (19.3)	9 (8.9)	0.024
Duration of high-flow nasal	2 (1-3)	2 (1-3)	2 (1-3)	>0.999**

oxygen[b]				
Additional ICU support, no. (%)				
Vasopressors/inotropes	163 (64.7)	82 (54.3)	81 (80.2)	<0.001
Renal replacement therapy[b]	101 (40.2)	43 (28.7)	58 (57.4)	<0.001
Red blood cell transfusion[b]	93 (37.1)	48 (32.0)	45 (44.6)	0.043
Platelet transfusion[b]	50 (19.9)	20 (13.3)	30 (29.7)	0.001
Fresh frozen plasma transfusion[b]	58 (23.1)	28 (18.7)	30 (29.7)	0.042
Surgical source control[b]	25 (10.0)	19 (12.7)	6 (5.9)	0.081
Non-surgical source control[b]	78 (31.1)	54 (36.0)	24 (23.8)	0.040
**Outcomes**				
Patient status, no. (%)				<0.001*
Alive upon current hospital discharge	150 (59.5)	150 (99.3)	0 (0.0)	
Alive upon discharge from current ICU stay, but died in current hospital stay	17 (6.7)	0 (0.0)	17 (16.8)	
Alive upon discharge from current ICU stay, but still in current hospital stay after 90 days	1 (0.4)	1 (0.7)	0 (0.0)	
Still in current ICU stay after 90 days	0 (0.0)	0 (0.0)	0 (0.0)	
Died in current ICU stay	84 (33.3)	0 (0.0)	84 (83.2)	
Length of stay, median days (Q1-Q3)				
Hospital	16 (10-25)	17 (11-24)	13 (7-26)	0.027**
ICU	10 (6-18)	10.5 (6-17)	10 (5-21)	0.740**

[a]Comparison between the patients who survived and died using Chi-squared test; *Fisher's exact test; **Mann–Whitney U test.

[b]One missing patient

Abbreviations

ICU: intensive care unit; **n**, total number of patients; **no.**, total number of patients recorded if a variable was given; **Q**: quartile

Table S3. Clinical, laboratory and microbiology characteristics and severity of illness upon intensive care unit (ICU) admission according to ICU survivability of patients with sepsis

Variable	All cases n=252	Survived n=168	Died n=84	p-value[a]
Participating hospital, no. (%)				
115 People's	25 (9.9)	7 (4.2)	18 (21.4)	
Bach Mai[b]	26 (10.3)	16 (9.5)	10 (11.9)	
Bai Chay	14 (5.6)	10 (6.0)	4 (4.8)	
Can Tho[b]	7 (2.8)	4 (2.4)	3 (3.6)	
Cho Ray[b]	41 (16.3)	22 (13.1)	19 (22.6)	
Da Nang	12 (4.8)	6 (3.6)	6 (7.1)	
Dong Da	9 (3.6)	6 (3.6)	3 (3.6)	
Hanoi Medical University	12 (4.8)	6 (3.6)	6 (7.1)	
Hue[b]	39 (15.5)	31 (18.5)	8 (9.5)	
Saint Paul	9 (3.6)	9 (5.4)	0 (0.0)	
Thai Nguyen[b]	2 (0.8)	1 (0.6)	1 (1.2)	
Thanh Nhan	1 (0.4)	1 (0.6)	0 (0.0)	
Vietnam–Czechoslovakia Friendship	48 (19.0)	42 (25.0)	6 (7.1)	
Vinmec Times City International	7 (2.8)	7 (4.2)	0 (0.0)	
Age (year), median (Q1-Q3)	65 (52-77)	65 (52-76)	65 (52-77)	0.971**
Age (year), no. (%)				0.844*
< 20	3 (1.2)	2 (1.2)	1 (1.2)	
20 - 39	19 (7.5)	11 (6.5)	8 (9.5)	
40 - 59	74 (29.4)	49 (29.2)	25 (29.8)	
≥ 60	156 (61.9)	106 (63.1)	50 (59.5)	
Age (year), no. (%)				>0.999
< 65	123 (48.8)	82 (48.8)	41 (48.8)	
≥ 65	129 (51.2)	86 (51.2)	43 (51.2)	
Sex (male), no. (%)	162 (64.3)	104 (61.9)	58 (69.0)	0.265
Comorbidities, no. (%)				
Cardiovascular disease	78 (31.0)	47 (28.0)	31 (36.9)	0.148

Chronic lung disease	30 (11.9)	21 (12.5)	9 (10.7)	0.680
Chronic neurological disease	36 (14.3)	28 (16.7)	8 (9.5)	0.127
Chronic kidney disease	23 (9.1)	16 (9.5)	7 (8.3)	0.757
Peptic ulcer disease	9 (3.6)	6 (3.6)	3 (3.6)	>0.999*
Chronic liver disease	27 (10.7)	17 (10.1)	10 (11.9)	0.670
Diabetes mellitus	67 (26.6)	44 (26.2)	23 (27.4)	0.840
HIV infection	0 (0.0)	0 (0.0)	0 (0.0)	NA
Connective tissue disease	3 (1.2)	2 (1.2)	1 (1.2)	>0.999*
Immunosuppression	10 (4.0)	7 (4.2)	3 (3.6)	>0.999*
Hematological malignancies	5 (2.0)	3 (1.8)	2 (2.4)	>0.999*
Solid malignant tumors	12 (4.8)	6 (3.6)	6 (7.1)	0.222*
**Vital signs** (on admission into ICU)				
GCS, median (Q1-Q3)	13 (9-15)	14 (10-15)	10 (8-14)	<0.001**
HR (beats per min), median (Q1-Q3)	110 (95-126)	109 (92-121)	112 (100-130)	0.008**
Temperature (°C), mean (SD)	37.79 (1.01)	37.82 (1.07)	37.72 (0.88)	0.485**
MBP (mmHg), mean(SD)	75.82 (22.08)	78.95 (22.80)	69.54 (19.21)	0.001**
SBP (mmHg), mean (SD)	106.45 (29.96)	110.64 (29.48)	98.08 (29.33)	0.002**
RR (breaths per min), median (Q1-Q3)	25 (22-30)	25 (22-30)	25 (20-30)	>0.999**
**Blood investigations**				
Total WBC ($x10^9$/L), mean (SD)	15.73 (9.20)	15.70 (8.64)	15.79 (10.28)	0.941**
PLT ($x10^9$/L), mean (SD)	185.98 (137.85)	203.72 (131.99)	150.49 (143.17)	0.004**
Hb (g/dL), mean (SD)	11.14 (2.59)	11.33 (2.62)	10.77 (2.50)	0.104**
Hct (%), mean (SD)	34.31 (7.75)	34.85 (7.76)	33.24 (7.67)	0.122**
K^+ (mmol/L), mean (SD)	3.89 (0.79)	3.89 (0.80)	3.87 (0.77)	0.838**
Na^+ (mmol/L), mean (SD)	136.05 (8.24)	135.21 (8.72)	137.74 (6.92)	0.021**
Creatinine (µmol/L), mean (SD)	187.85	188.47	186.60	0.927**

	(151.92)	(169.24)	(110.29)	
Bilirubin (µmol/l), mean (SD)	32.80 (61.49)	31.40 (69.33)	35.52 (42.65)	0.629[**]
pH, mean (SD)	7.37 (0.50)	7.40 (0.61)	7.32 (0.13)	0.249[**]
PaO_2 (mmHg), mean (SD)	116.17 (74.28)	111.80 (64.31)	124.49 (90.14)	0.206[**]
FiO_2, mean (SD)	0.50 (0.22)	0.45 (0.20)	0.57 (0.24)	<0.001[**]
PaO_2/FiO_2 ratio, mean (SD)	262.48 (149.58)	273.45 (149.45)	241.73 (148.49)	0.116[**]
**Severity of illness scores**				
qSOFA, median (Q1-Q3)	2 (1-2)	2 (1-2)	2 (2-3)	0.001[**]
qSOFA, no. (%)				0.003
0	9 (3.6)	6 (3.6)	3 (3.6)	
1	60 (23.8)	47 (28.0)	13 (15.5)	
2	124 (49.2)	87 (51.8)	37 (44.0)	
3	59 (23.4)	28 (16.7)	31 (36.9)	
qSOFA, no. (%)				0.036
0 - 1	69 (27.4)	53 (31.5)	16 (19.0)	
2 - 3	183 (72.6)	115 (68.5)	68 (81.0)	
qSOFA, no. (%)				<0.001
0 - 2	193 (76.6)	140 (83.3)	53 (63.1)	
3	59 (23.4)	28 (16.7)	31 (36.9)	
SIRS criteria, median (Q1-Q3)	3 (2-4)	3 (3-4)	3 (2-4)	0.792[**]
SOFA score, median (Q1-Q3)	7 (5-10)	6 (4-9)	10 (7-13)	<0.001[**]
SOFA score, no. (%)				<0.001
< 8	140 (56.0)	113 (68.1)	27 (32.1)	
≥ 8	110 (44.0)	53 (31.9)	57 (67.9)	
APACHE II score, median (Q1-Q3)	18 (13-24)	16 (12-22)	22 (16-27)	<0.001[**]
APACHE II score, no. (%)				<0.001
< 19	129 (51.2)	103 (61.3)	26 (31.0)	
≥ 19	123 (48.8)	65 (38.7)	58 (69.0)	
Sheptic Shock, no. (%)	74 (29.4)	43 (25.6)	31 (36.9)	0.063

**Site of Infection**				
Respiratory, no. (%)	143 (56.7)	93 (55.4)	50 (59.5)	0.529
Urinary tract, no. (%)	37 (14.7)	31 (18.5)	6 (7.1)	0.017
Abdominal, no. (%)	61 (24.2)	37 (22.0)	24 (28.6)	0.253
Neurological, no. (%)	12 (4.8)	9 (5.4)	3 (3.6)	0.756*
Bones or joints, no. (%)	2 (0.8)	2 (1.2)	0 (0.0)	0.554*
Skin or cutaneous sites, no. (%)	19 (7.5)	9 (5.4)	10 (11.9)	0.063
Intravascular catheter, no. (%)	1 (0.4)	1 (0.6)	0 (0.0)	>0.999*
Infective endocarditis, no. (%)	1 (0.4)	0 (0.0)	1 (1.2)	0.333*
Primary bacteremia, no. (%)	7 (2.8)	6 (3.6)	1 (.2)	0.430*
Systemic, no. (%)	6 (2.4)	4 (2.4)	2 (2.4)	>0.999*
**Microbiology**				
No pathogens detected, no. (%)	67 (26.6)	50 (29.8)	17 (20.2)	0.107
Gram negative bacteria, no. (%)	156 (61.9)	101 (60.1)	55 (65.5)	0.409
Klebsiella pneumonia	27 (10.7)	17 (10.1)	10 (11.9)	0.666
Acinetobacter baumannii	45 (17.9)	24 (14.3)	21 (25.0)	0.036
Escherichia coli	44 (17.5)	31 (18.5)	13 (15.5)	0.557
Pseudomonas aeruginosa	24 (9.5)	18 (10.7)	6 (7.1)	0.363
Stenotrophomonas maltophilia	2 (0.8)	1 (0.6)	1 (1.2)	>0.999*
Proteus species	47 (18.7)	30 (17.9)	17 (20.2)	0.647
Enterobacter cloacae	3 (1.2)	3 (1.8)	0 (0.0)	0.553*
Bulkholderia pseudomallei	1 (0.4)	1 (0.6)	0 (0.0)	>0.999
Gram positive bacteria, no. (%)	34 (13.5)	23 (13.7)	11 (13.1)	0.896
Enterococcus	5 (2.0)	5 (3.0)	0 (0.0)	0.173*
MSSA	5 (2.0)	3 (1.8)	2 (2.4)	>0.999*
MRSA	10 (4.0)	7 (4.2)	3 (3.6)	>0.999*
Other Streptococcus species	12 (4.8)	6 (3.6)	6 (7.1)	0.222*
Streptococcus pneumonia	2 (0.8)	2 (1.2)	0 (0.0)	0.554*
Fungi, no. (%)	7 (2.8)	4 (2.4)	3 (3.6)	0.689*
Candida species	7 (2.8)	4 (2.4)	3 (3.6)	0.689*
Aspergillus species	0 (0.0)	0 (0.0)	0 (0.0)	NA
Viruses, no. (%)	2 (0.8)	0 (0.0)	2 (2.4)	0.110*

Influenza	1 (0.4)	0 (0.0)	1 (1.2)	0.333*
Dengue	1 (0.4)	0 (0.0)	1 (1.2)	0.333*
Other pathogens, no. (%)				
Anaerobes	0 (0.0)	0 (0.0)	0 (0.0)	NA
Mycobacterium tuberculosis	4 (1.6)	3 (1.8)	1 (1.2)	>0.999*
Malaria	0 (0.0)	0 (0.0)	0 (0.0)	NA

[a]Comparison between the patients who survived and died using Chi-squared test; *Fisher's exact test; **Mann–Whitney U test.

[b]Central hospitals

Abbreviations

APACHE II score: Acute Physiology and Chronic Health Evaluation II score; **FiO_2**: fraction of inspired oxygen; **GCS**: Glasgow Coma Scale; **Hb**: hemoglobin; **Hct**: hematocrit; **HIV**: human immunodeficiency virus; **HR**: heart rate; **ICU**: intensive care unit; **MBP**: mean arterial blood pressure; **n**, total number of patients; **NA**, not available; **no.**, total number of patients recorded if a variable was given; **MRSA**: methicillin-resistant Staphylococcus aureus; **MSSA**: methicillin-susceptible Staphylococcus aureus; **PaO_2**: partial pressure of oxygen in the arterial blood; **PLT**: platelet; **Q**: quartile; **qSOFA score**: quick Sequential (Sepsis-Related) Organ Failure Assessment score; **RR**: respiration rate; **SBP**: systolic blood pressure; **SD**: standard deviation; **SIRS criteria**: Systemic Inflammatory Response Syndrome criteria; **SOFA score**: Sequential (Sepsis-Related) Organ Failure Assessment score; **WBC**: white blood cell.

Table S4. Life-sustaining treatments during intensive care unit (ICU) stay and outcomes according to ICU survivability of patients with sepsis

Variable	All cases n=252	Survived n=168	Died n=84	p-value[a]
**Life-sustaining treatments during ICU stay**				
Respiratory support, no. (%) or median (Q1-Q3) days				
Mechanical ventilation[b]	173 (68.9)	97 (58.1)	76 (90.5)	<0.001
Duration of mechanical ventilation[b]	8 (4-15)	9 (4-15)	7 (3-15)	0.502**
Non-invasive ventilation[b]	20 (8.0)	14 (8.4)	6 (7.1)	0.732
Duration of non-invasive ventilation[b]	2 (2-4)	2 (1-2)	5 (4-11)	0.002**
High-flow nasal oxygen[b]	38 (15.1)	33 (19.8)	5 (6.0)	0.004

Duration of high-flow nasal oxygen[b]	2 (1-3)	2 (1-3)	3 (2-11)	0.146
Additional ICU support, no. (%)				
Vasopressors/inotropes	163 (64.7)	96 (57.1)	67 (79.8)	<0.001
Renal replacement therapy[b]	101 (40.2)	48 (28.7)	43 (63.1)	<0.001
Red blood cell transfusion[b]	93 (37.1)	55 (32.9)	38 (45.2)	0.057
Platelet transfusion[b]	50 (19.9)	23 (13.8)	27 (32.1)	0.001
Fresh frozen plasma transfusion[b]	58 (23.1)	32 (19.2)	26 (31.0)	0.037
Surgical source control[b]	25 (10.0)	19 (11.4)	6 (7.1)	0.290
Non-surgical source control[b]	78 (31.1)	59 (35.3)	19 (22.6)	0.040
Length of of surgical source control, median (Q1-Q3) minutes	290 (105-630)	290 (105-630)	430 (270-1587)	0.241**
**Outcomes**				
Patient status, no. (%)				<0.001*
Alive upon current hospital discharge	150 (59.5)	150 (89.3)	0 (0.0)	
Alive upon discharge from current ICU stay, but died in current hospital stay	17 (6.7)	17 (10.1)	0 (0.0)	
Alive upon discharge from current ICU stay, but still in current hospital stay after 90 days	1 (0.4)	1 (0.6)	0 (0.0)	
Still in current ICU stay after 90 days	0 (0.0)	0 (0.0)	0 (0.0)	
Died in current ICU stay	84 (33.3)	0 (0.0)	84 (100)	
Mortality, no. (%)				
Hospital	101 (40.1)	17 (10.1)	84 (100)	<0.001
Length of stay, median (Q1-Q3) days				
Hospital	16 (10-25)	17 (11-26)	13 (6-22)	0.002**
ICU	10 (6-18)	10 (6-17)	10 (5-21)	0.688**

[a]Comparison between the patients who survived and died using Chi-squared test; *Fisher's exact test; **Mann–

Whitney U test.

[b]One missing patient

Abbreviations

ICU: intensive care unit; **n**, total number of patients; **no.**, total number of patients recorded if a variable was given; **Q**: quartile

Table S5. Factors associated with hospital mortality in patients with sepsis upon intensive care unit admission: univariable regression analyses

Factor	Number of patients	OR	95.0% CI for OR		p-value
			Lower	Upper	
**Baseline characteristics**					
Central hospitals[a]	115	1.695	1.020	2.819	0.042
Age (year)	252	1.001	0.986	1.016	0.921
Age (year) group					
< 20	3				0.863
20 - 39	19	1.800	0.139	23.374	0.653
40 - 59	74	1.442	0.125	16.617	0.769
≥ 60	156	1.250	0.111	14.086	0.857
Age ≥ 65 years	129	1.020	0.616	1.688	0.939
Sex (male)	162	1.345	0.790	2.290	0.275
Documented comorbidities					
Cardiovascular disease	78	1.551	0.903	2.664	0.112
Chronic lung disease	30	0.996	0.458	2.169	0.992
Chronic neurological disease	36	0.378	0.165	0.867	0.022
Chronic kidney disease	23	0.957	0.398	2.304	0.922
Peptic ulcer disease	9	1.204	0.315	4.597	0.786
Chronic liver disease	27	1.446	0.649	3.220	0.367
Diabetes mellitus	67	1.012	0.573	1.790	0.966
Connective tissue disease	3	0.745	0.067	8.326	0.811
Immunosuppression	10	0.630	0.159	2.495	0.510
Hematological malignancies	5	0.997	0.164	6.073	0.997
Solid malignant tumors	12	1.526	0.478	4.873	0.475
**Vital signs**					

GCS	251	0.849	0.786	0.918	<0.001
HR (beats per min)	252	1.010	0.997	1.022	0.140
Temperature (^oC)	252	0.975	0.760	1.252	0.845
MBP (mmHg)	252	0.978	0.965	0.991	0.001
SBP (mmHg)	252	0.986	0.977	0.995	0.002
RR (breaths per min)	252	0.987	0.944	1.031	0.555
**Blood investigations**					
Total WBC ($x10^9$/L)	252	1.003	0.976	1.031	0.833
PLT ($x10^9$/L)	252	0.998	0.996	1.000	0.040
Hb (g/dL)	251	0.922	0.835	1.018	0.109
Hct (%)	252	0.968	0.936	1.001	0.057
K^+ (mmol/L)	252	0.961	0.697	1.324	0.805
Na^+ (mmol/L)	252	1.016	0.985	1.048	0.313
Creatinine (µmol/L)	252	1.000	0.999	1.002	0.828
Bilirubin (µmol/l)	232	1.001	0.996	1.005	0.752
pH	248	0.045	0.005	0.389	0.005
PaO_2 (mmHg)	244	1.003	0.999	1.006	0.142
FiO_2	245	26.892	7.081	102.133	<0.001
PaO_2/FiO_2 ratio	243	0.998	0.996	1.000	0.020
**Severity of illness scores**					
qSOFA score	252	1.697	1.203	2.393	0.003
qSOFA score					
0	9	-	-	-	0.007
1	60	0.857	0.193	3.810	0.840
2	124	1.139	0.272	4.777	0.859
3	59	2.917	0.664	12.813	0.156
qSOFA score					
0 - 1	69				
2 - 3	183	1.775	0.984	3.203	0.057
qSOFA score					
0 - 2	193				
3	59	2.806	1.542	5.106	0.001

SIRS criteria	252	1.004	0.776	1.300	0.974
SOFA score	250	1.219	1.130	1.315	<0.001
SOFA score					
< 8	140				
≥ 8	110	4.173	2.440	7.137	<0.001
APACHE II score	252	1.088	1.050	1.127	<0.001
APACHE II score					
< 19	129				
≥ 19	123	3.873	2.268	6.615	<0.001
**Site of Infection**					
Respiratory	143	1.283	0.769	2.140	0.339
Urinary tract	37	0.300	0.126	0.714	0.006
Abdominal	61	1.256	0.701	2.249	0.444
Neurological	12	0.737	0.216	2.516	0.626
Bones or joints	2	0.000	0.000	-	0.999
Skin or cutaneous sites	19	2.774	1.053	7.309	0.039
Intravascular catheter	1	0.000	0.000	-	1.000
Infective endocarditis	1	2439367045	0.000	-	>0.999
Primary bacteraemia	7	0.590	0.112	3.101	0.533
Systemic	6	0.742	0.133	4.131	0.734

[b]Central hospitals included the Thai Nguyen, Bach Mai, Hue, Cho Ray, and Can Tho hospitals.

Abbreviations

APACHE II score: Acute Physiology and Chronic Health Evaluation II score; **CI**: confidence interval; **FiO_2**: fraction of inspired oxygen; **GCS**: Glasgow Coma Scale; **Hb**: hemoglobin; **Hct**: hematocrit; **HR**: heart rate; **MBP**: mean arterial blood pressure; **OR**: odds ratio; **PaO_2**: partial pressure of oxygen in the arterial blood; **PLT**: platelet; **qSOFA score**: quick Sequential (Sepsis-Related) Organ Failure Assessment score; **RR**: respiration rate; **SBP**: systolic blood pressure; **SIRS criteria**: Systemic Inflammatory Response Syndrome criteria; **SOFA score**: Sequential (Sepsis-Related) Organ Failure Assessment score; **WBC**: white blood cell.

Table S6. Factors associated with intensive care unit (ICU) mortality in patients with sepsis upon ICU admission: univariable regression analyses

Factor	Number of patients	OR	95.0% CI for OR		p-value
			Lower	Upper	

Central hospitals[a]	115	1.211	0.716	2.048	0.475
**Baseline characteristics**					
Age (year)	252	0.998	0.983	1.014	0.802
Age (year) group					
< 20	3				0.856
20 - 39	19	1.455	0.112	18.956	0.775
40 - 59	74	1.020	0.088	11.805	0.987
≥ 60	156	0.943	0.084	10.651	0.962
Age ≥ 65 years	129	1.000	0.592	1.689	>0.999
Sex (male)	162	0.728	0.417	1.272	0.265
Documented comorbidities					
Cardiovascular disease	78	1.506	0.863	2.627	0.150
Chronic lung disease	30	0.840	0.367	1.924	0.680
Chronic neurological disease	36	0.526	0.229	1.212	0.131
Chronic kidney disease	23	0.864	0.341	2.188	0.757
Peptic ulcer disease	9	1.000	0.244	4.101	>0.999
Chronic liver disease	27	1.200	0.524	2.750	0.666
Diabetes mellitus	67	1.063	0.589	1.917	0.840
Connective tissue disease	3	1.000	0.089	11.188	>0.999
Immunosuppression	10	0.852	0.215	3.381	0.820
Hematological malignancies	5	1.341	0.220	8.186	0.750
Solid malignant tumors	12	2.077	0.649	6.648	0.218
**Vital signs**					
GCS	251	0.589	0.794	0.930	<0.001
HR (beats per min)	252	1.017	1.003	1.030	0.014
Temperature (^{o}C)	252	0.910	0.700	1.184	0.484
MBP (mmHg)	252	0.978	0.965	0.992	0.002
SBP (mmHg)	252	0.985	0.976	0.995	0.002
RR (breaths per min)	252	0.993	0.949	1.040	0.774
**Blood investigations**					
Total WBC (x10^9/L)	252	1.001	0.973	1.030	0.941
PLT (x10^9/L)	252	0.997	0.994	0.999	0.005

Hb (g/dL)	251	0.918	0.828	1.018	0.105
Hct (%)	252	0.973	0.940	1.007	0.123
K^+ (mmol/L)	252	0.966	0.692	1.348	0.837
Na^+ (mmol/L)	252	1.038	1.005	1.073	0.025
Creatinine (µmol/L)	252	1.000	0.998	1.002	0.926
Bilirubin (µmol/l)	232	1.001	0.997	1.005	0.631
pH	248	0.131	0.016	1.108	0.062
PaO_2 (mmHg)	244	1.002	0.999	1.006	0.212
FiO_2	245	11.704	3.405	40.224	<0.001
PaO_2/FiO_2 ratio	243	0.998	0.997	1.000	0.119
**Severity of illness scores**					
qSOFA score	252	1.768	1.231	2.540	0.002
qSOFA score					
0	9	-	-	-	0.004
1	60	0.553	0.121	2.519	0.444
2	124	0.851	0.202	3.584	0.825
3	59	2.214	0.506	9.699	0.292
qSOFA score					
0 - 1	69				
2 - 3	183	1.959	1.039	3.694	0.038
qSOFA score					
0 - 2	193				
3	59	2.925	1.604	5.333	<0.001
SIRS criteria	252	1.045	0.798	1.368	0.750
SOFA score	250	1.244	1.149	1.346	<0.001
SOFA score					
< 8	140				
≥ 8	110	4501	2565	7.898	<0.001
APACHE II score	252	1.076	1.039	1.114	<0.001
APACHE II score					
< 19	129				
≥ 19	123	3.535	2.025	6.171	<0.001

Site of Infection					
Respiratory	143	1.186	0.697	2.018	0.529
Urinary tract	37	0.340	0.136	0.851	0.021
Abdominal	61	1.416	0.779	2.575	0.254
Neurological	12	0.654	0.172	2.483	0.533
Bones or joints	2	0.000	0.000	-	0.999
Skin or cutaneous sites	19	2.387	0.931	6.123	0.070
Intravascular catheter	1	0.000	0.000	-	>0.999
Infective endocarditis	1	3269876834	0.000	-	>0.999
Primary bacteraemia	7	0.325	0.039	2.747	0.302
Systemic	6	1.000	0.179	5.573	>0.999

[a]Central hospitals included the Thai Nguyen, Bach Mai, Hue, Cho Ray, and Can Tho hospitals.

Abbreviations

APACHE II score: Acute Physiology and Chronic Health Evaluation II; **CI**: confidence interval; **FiO_2**: fraction of inspired oxygen; **GCS**: Glasgow Coma Scale; **Hb**: hemoglobin; **Hct**: hematocrit; **HR**: heart rate; **MBP**: mean arterial blood pressure; **OR**: odds ratio; **PaO_2**: partial pressure of oxygen in the arterial blood; **PLT**: platelet; **qSOFA score**: quick Sequential (Sepsis-Related) Organ Failure Assessment score; **RR**: respiration rate; **SBP**: systolic blood pressure; **SIRS criteria**: Systemic Inflammatory Response Syndrome criteria; **SOFA score**: Sequential (Sepsis-Related) Organ Failure Assessment score; **WBC**: white blood cell.

Table S7. Factors (including the qSOFA score of 3) associated with hospital mortality in patients with sepsis upon intensive care unit admission: multivariable logistic regression analyses (backward elimination)

Step	Factor	Number of patients	AOR	95.0% CI for AOR		p-value
				Lower	Upper	
1	Central hospitals[b]	115	2.318	1.241	4.329	0.008
	Age of 65 years or older	129	1.223	0.647	2.312	0.535
	Sex (male)	162	1.702	0.935	3.099	0.082
	Cardiovascular disease	78	2.051	1.041	4.044	0.038
	Chronic neurological disease	36	0.409	0.159	1.053	0.064
	Solid malignant tumors	12	2.062	0.520	8.183	0.303
	Urinary tract	37	0.266	0.104	0.678	0.006
	Skin or cutaneous Sites	19	3.134	1.083	9.069	0.035
	qSOFA score of 3	183	3.656	1.877	7.122	<0.001
	Constant		0.195			<0.001
2	Central hospitals[b]	115	2.226	1.209	4.098	0.010

	Sex (male)	162	1.702	0.935	3.099	0.082
	Cardiovascular disease	78	2.051	1.041	4.044	0.038
	Chronic neurological disease	36	0.409	0.159	1.053	0.064
	Solid malignant tumors	12	2.062	0.520	8.183	0.303
	Urinary tract	37	0.266	0.104	0.678	0.006
	Skin or cutaneous Sites	19	3.134	1.083	9.069	0.035
	qSOFA score of 3	183	3.610	1.858	7.015	<0.001
	Constant		0.217			<0.001
3	Central hospitals[b]	115	2.062	1.145	3.714	0.016
	Sex (male)	162	1.644	0.909	2.974	0.100
	Cardiovascular disease	78	2.118	1.138	3.941	0.018
	Chronic neurological disease	36	0.399	0.157	1.017	0.054
	Urinary tract	37	0.268	0.105	0.683	0.006
	Skin or cutaneous sites	19	3.000	1.047	8.597	0.041
	qSOFA score of 3	183	3.613	1.866	6.995	<0.001
	Constant		0.240			<0.001
4[a]	Central hospitals[b]	115	1.992	1.110	3.573	0.021
	Cardiovascular disease	78	2.011	1.086	3.721	0.026
	Chronic neurological disease	36	0.387	0.152	0.983	0.046
	Urinary tract	37	0.270	0.106	0.686	0.006
	Skin or cutaneous sites	19	3.013	1.059	8.571	0.039
	qSOFA score of 3	183	3.358	1.756	6.422	<0.001
	Constant		0.350			<0.001

[a]Step 4 is the final multivariable logistic regression model; [b]Central hospitals included the Thai Nguyen, Bach Mai, Hue, Cho Ray, and Can Tho hospitals.
Abbreviations: **CI**: confidence interval; **AOR**: adjusted odds ratio; **qSOFA score**: quick Sequential (Sepsis-Related) Organ Failure Assessment score.

Table S8. Factors (including the qSOFA score of 3) associated with intensive care unit (ICU) mortality in patients with sepsis upon ICU admission: multivariable logistic regression analyses (backward elimination)

Step	Factor	Number of patients	AOR	95.0% CI for AOR		p-value
				Lower	Upper	
1	Central hospitals[b]	115	1.542	0.824	2.887	0.176
	Age of 65 years or older	129	1.023	0.535	1.957	0.944
	Sex (male)	162	1.671	0.907	3.080	0.100
	Cardiovascular disease	78	1.922	0.971	3.804	0.061
	Chronic neurological disease	36	0.556	0.217	1.428	0.223

	Solid malignant tumors	12	2.472	0.642	9.528	0.188
	Urinary tract	37	0.331	0.126	0.874	0.026
	Skin or cutaneous Sites	19	2.460	0.887	6.824	0.084
	qSOFA score of 3	183	3.459	1.810	6.609	<0.001
	Constant		0.185			<0.001
2	Central hospitals[b]	115	1.535	0.831	2.837	0.171
	Sex (male)	162	1.668	0.908	3.064	0.099
	Cardiovascular disease	78	1.940	1.035	3.638	0.039
	Chronic neurological disease	36	0.558	0.218	1.427	0.223
	Solid malignant tumors	12	2.477	0.644	9.530	0.187
	Urinary tract	37	0.332	0.126	0.874	0.026
	Skin or cutaneous Sites	19	2.458	0.886	6.815	0.084
	qSOFA score of 3	183	3.455	1.810	6.597	<0.001
	Constant		0.187			<0.001
3	Central hospitals[b]	115	1.715	0.950	3.098	0.074
	Sex (male)	162	1.694	0.925	3.100	0.088
	Cardiovascular disease	78	1.803	0.975	3.336	0.060
	Chronic neurological disease	36	2.762	0.722	10.560	0.138
	Urinary tract	37	0.318	0.121	0.837	0.020
	Skin or cutaneous sites	19	2.656	0.970	7.272	0.057
	qSOFA score of 3	183	3.399	1.789	6.461	<0.001
	Constant		0.167			<0.001
4	Central hospitals[b]	115	1.568	0.885	2.780	0.124
	Sex (male)	162	1.677	0.918	3.062	0.093
	Cardiovascular disease	78	1.675	0.917	3.058	0.093
	Urinary tract	37	0.313	0.119	0.823	0.018
	Skin or cutaneous sites	19	2.571	0.951	6.951	0.063
	qSOFA score of 3	183	3.409	1.804	6.442	<0.001
	Constant		0.189			<0.001
5	Sex (male)	162	1.652	0.906	3.011	0.101
	Cardiovascular disease	78	1.566	0.866	2.831	0.138
	Urinary tract	37	0.334	0.129	0.864	0.024
	Skin or cutaneous sites	19	2.343	0.875	6.273	0.090
	qSOFA score of 3	183	3.289	1.750	6.182	<0.001
	Constant		0.243			<0.001
6	Sex (male)	162	1.556	0.862	2.806	0.142
	Urinary tract	37	0.316	0.122	0.819	0.018
	Skin or cutaneous sites	19	2.345	0.879	6.251	0.089
	qSOFA score of 3	183	3.236	1.728	6.061	<0.001
	Constant		0.296			<0.001

7[a]	Urinary tract	37	0.318	0.123	0.822	0.018
	Skin or cutaneous sites	19	2.365	0.893	6.264	0.083
	qSOFA score of 3	183	3.060	1.651	5.671	<0.001
	Constant		0.400			<0.001

[a]Step 7 is the final multivariable logistic regression model; [b]Central hospitals included the Thai Nguyen, Bach Mai, Hue, Cho Ray, and Can Tho hospitals.

Abbreviations: **CI**: confidence interval; **AOR**: adjusted odds ratio; **qSOFA score**: quick Sequential (Sepsis-Related) Organ Failure Assessment score.

Table S9. Factors (including the qSOFA score of 2 to 3) associated with hospital mortality in patients with sepsis upon intensive care unit admission: multivariable logistic regression analyses (backward elimination)

Step	Factor	Number of patients	AOR	95.0% CI for AOR		p-value
				Lower	Upper	
1	Central hospitals[b]	115	2.144	1.162	3.955	0.015
	Age of 65 years or older	129	1.144	0.613	2.132	0.673
	Sex (male)	162	1.640	0.910	2.955	0.100
	Cardiovascular disease	78	2.138	1.092	4.185	0.027
	Chronic neurological disease	36	0.380	0.148	0.973	0.044
	Solid malignant tumors	12	2.261	0.607	8.431	0.224
	Urinary tract	37	0.294	0.118	0.735	0.009
	Skin or cutaneous Sites	19	2.886	1.029	8.097	0.044
	qSOFA score of 2 to 3	183	2.321	1.216	4.431	0.011
	Constant		0.156			<0.001
2	Central hospitals[b]	115	2.087	1.146	3.798	0.016
	Sex (male)	162	1.616	0.901	2.900	0.108
	Cardiovascular disease	78	2.254	1.205	4.215	0.011
	Chronic neurological disease	36	0.386	0.151	0.987	0.047
	Solid malignant tumors	12	2.289	0.617	8.490	0.216
	Urinary tract	37	0.298	0.119	0.743	0.009
	Skin or cutaneous Sites	19	2.876	1.025	8.074	0.045
	qSOFA score of 2 to 3	183	2.321	1.216	4.431	0.011
	Constant		0.167			<0.001
3	Central hospitals[b]	115	1.912	1.073	3.405	0.028
	Sex (male)	162	1.599	0.892	2.866	0.115
	Cardiovascular disease	78	2.138	1.155	3.958	0.016
	Chronic neurological disease	36	0.363	0.143	0.923	0.033
	Urinary tract	37	0.298	0.120	0.742	0.009
	Skin or cutaneous sites	19	2.802	1.005	7.807	0.049
	qSOFA score of 2 to 3	183	2.283	1.198	4.350	0.012
	Constant		0.190			<0.001

4[a]	Central hospitals[b]	115	1.853	1.045	3.286	0.035
	Cardiovascular disease	78	2.031	1.105	3.734	0.023
	Chronic neurological disease	36	0.348	0.137	0.885	0.027
	Urinary tract	37	0.304	0.123	0.751	0.010
	Skin or cutaneous sites	19	2.847	1.022	7.928	0.045
	qSOFA score of 2 to 3	183	2.101	1.118	3.951	0.021
	Constant		0.282			<0.001

[a]Step 4 is the final multivariable logistic regression model; [b]Central hospitals included the Thai Nguyen, Bach Mai, Hue, Cho Ray, and Can Tho hospitals.

Abbreviations: **CI**: confidence interval; **AOR**: adjusted odds ratio; **qSOFA score**: quick Sequential (Sepsis-Related) Organ Failure Assessment score.

Table S10. Factors (including the qSOFA score of 2 to 3) associated with intensive care unit (ICU) mortality in patients with sepsis upon ICU admission: multivariable logistic regression analyses (backward elimination)

Step	Factor	Number of patients	AOR	95.0% CI for AOR		p-value
				Lower	Upper	
1	Central hospitals[b]	115	1.452	0.783	2.693	0.236
	Age of 65 years or older	129	0.962	0.509	1.820	0.906
	Sex (male)	162	1.623	0.891	2.959	0.114
	Cardiovascular disease	78	2.015	1.023	3.967	0.043
	Chronic neurological disease	36	0.501	0.196	1.284	0.150
	Solid malignant tumors	12	2.758	0.754	10.093	0.125
	Urinary tract	37	0.360	0.139	0.934	0.036
	Skin or cutaneous Sites	19	2.302	0.854	6.205	0.099
	qSOFA score of 2 to 3	183	2.436	1.237	4.794	0.010
	Constant		0.140			<0.001
2	Central hospitals[b]	115	1.463	0.799	2.680	0.218
	Sex (male)	162	1.629	0.896	2.961	0.109
	Cardiovascular disease	78	1.982	1.064	3.695	0.031
	Chronic neurological disease	36	0.499	0.195	1.276	0.147
	Solid malignant tumors	12	2.746	0.751	10.037	0.127
	Urinary tract	37	0.359	0.138	0.930	0.035
	Skin or cutaneous Sites	19	2.304	0.855	6.209	0.099
	qSOFA score of 2 to 3	183	2.434	1.237	4.789	0.010
	Constant		0.137			<0.001
3	Sex (male)	162	1.590	0.877	2.879	0.126
	Cardiovascular disease	78	1.911	1.031	3.542	0.040
	Chronic neurological disease	36	0.422	0.172	1.038	0.060
	Solid malignant tumors	12	2.261	0.654	7.815	0.197
	Urinary tract	37	0.376	0.146	0.969	0.043

	Skin or cutaneous Sites	19	2.094	0.788	5.568	0.138
	qSOFA score of 2 to 3	183	2.406	1.227	4.718	0.011
	Constant		0.176			<0.001
4	Sex (male)	162	1.583	0.875	2.864	0.129
	Cardiovascular disease	78	1.831	0.994	3.371	0.052
	Chronic neurological disease	36	0.410	0.167	1.005	0.051
	Urinary tract	37	0.371	0.145	0.953	0.039
	Skin or cutaneous sites	19	2.083	0.788	5.505	0.139
	qSOFA score of 2 to 3	183	2.370	1.211	4.639	0.012
	Constant		0.190			<0.001
5	Sex (male)	162	1.595	0.884	2.878	0.121
	Cardiovascular disease	78	1.847	1.005	3.395	0.048
	Chronic neurological disease	36	0.389	0.159	0.952	0.039
	Urinary tract	37	0.371	0.145	0.947	0.038
	qSOFA score of 2 to 3	183	2.403	1.234	4.682	0.010
	Constant		0.199			<0.001
6	Cardiovascular disease	78	1.752	0.959	3.199	0.068
	Chronic neurological disease	36	0.377	0.154	0.924	0.033
	Urinary tract	37	0.375	0.147	0.953	0.039
	qSOFA score of 2 to 3	183	2.222	1.153	4.282	0.017
	Constant		0.293			<0.001

[a]Step 6 is the final multivariable logistic regression model; [b]Central hospitals included the Thai Nguyen, Bach Mai, Hue, Cho Ray, and Can Tho hospitals.

Abbreviations: **CI**: confidence interval; **AOR**: adjusted odds ratio; **qSOFA score**: quick Sequential (Sepsis-Related) Organ Failure Assessment score.

Table S11. Breakdown of missing data

Variables	Number of patients with missing data
**Hospital characteristics**	
Type of hospital	0
University affiliation	0
**ICU characteristics**	
Nature of ICU	0
Type of ICU	0
Nurse to patient ratio	0
Intensivist to patient ratio	0
Training programme in ICU	0
**Baseline characteristics**	

Age (year)	0
Sex	0
Collection batch	0
Admission type	0
Admission source	0
Comorbidities	0
**Vital signs** (on admission into ICU)	
GCS	1
HR (beats per min)	0
Temperature (^{o}C)	0
MBP (mmHg)	0
SBP (mmHg)	0
RR (breaths per min)	0
**Blood investigations**	
Total WBC (x10^9/L)	0
PLT (x10^9/L)	0
Hb (g/dL)	1
Hct (%)	0
K^+ (mmol/L)	0
Na^+ (mmol/L)	0
Creatinine (µmol/L)	0
Bilirubin (µmol/l)	20
pH	3
PaO_2 (mmHg)	8
FiO_2 (mmHg)	7
PaO_2/FiO_2 ratio	9
**Severity of illness scores**	
qSOFA	0
SIRS	0
SOFA	2
APACHE II	0
**Site of Infection**	

Respiratory	0
Urinary tract	0
Abdominal	0
Neurological	0
Bones or joints	0
Skin or cutaneous sites	0
Intravascular catheter	0
Infective endocarditis	0
Primary bacteraemia	0
Systemic	0
**Measurements around time zero**	
Blood culture	0
Lactate measurement	0
Antibiotic administration	0
Fluid bolus	2
**Resources used in ICU** (anytime during ICU stay)	
Vasopressors/Intropes	0
Mechanical ventilation	1
Noninvasive ventilation	1
High-flow nasal cannula	1
Renal replacement therapy	1
Red blood cell transfusion	1
Platelet transfusion	1
Fresh frozen plasma transfusion	1
Surgical source control	1
Non-surgical source control	1
**In-hospital time course** (DD/MM/YY (HHMM))	
Admission date to the hospital	0
Admission date to the ICU	0
Time zero	0

Time of blood culture	2
Time of lactate measurement	1
Time of antibiotic administration	2
Time of starting vasopressor	3
Time of first source control measure	0
Discharge date from current ICU stay or death date in your current ICU stay	1
Discharge date from current hospital stay or death date in your current hospital stay	1
