# Supplementary material for: Predictive validity of the quick Sequential Organ Failure Assessment (qSOFA) score for the mortality in patients with sepsis in Vietnamese intensive care units
Source: PLoS One. 2022 Oct 14;17(10):e0275739. doi: 10.1371/journal.pone.0275739 (PMC9565713; doi:10.1371/journal.pone.0275739)
Supplement: Attachment — Submitted filename: Response to Reviewers.pdf [file pone.0275739.s007.pdf]

Professor Dinh-Toi Chu, PhD.
Academic Editor
PLOS ONE

August 21, 2022

Dear Prof. Dinh-Toi Chu,

On behalf of all authors, I am resubmitting herewith our revised manuscript entitled "Predictive validity of the quick Sequential Organ Failure Assessment (qSOFA) score for the mortality in patients with sepsis in Vietnamese intensive care units" (PONE-D-21-40686R1).

We sincerely appreciate the kind comments and points raised by the Editors and the Reviewers. We have carefully considered all comments and suggestions and revised our manuscript following these points. These comments have greatly enabled us to improve our manuscript. We hope that Editor will find our revised manuscript suitable for publication in PLOS ONE.

We confirm that this work is original and has not been published elsewhere, nor is it currently under consideration for publication elsewhere. All authors have read, approved the revised manuscript, and agreed to authorship and order of authorship for this manuscript. All authors have the appropriate permissions and rights to the reported data.

We have provided our point-by-point responses to the comments of the Editors and the Reviewers below.

We thank you for your kind consideration of this submission.

Sincerely yours,

Chinh Quoc Luong, MD., PhD.
Center for Emergency Medicine,
Bach Mai Hospital,
No. 78, Giai Phong, Phuong Mai ward, Dong Da district, Hanoi 100000, Vietnam
Email: luongquocchinh@gmail.com

We thank the Editors and the Reviewers for the valuable comments and suggestions that greatly helped us to improve the contents of this paper. In what follows, we will use the **boldface** to indicate comments from the Editors and the Reviewers, the standard font face for our responses and we highlighted in yellow the modifications that we did to the manuscript.

RESPONSE TO EDITORS

Dear Dr. Luong,

Thank you for submitting your manuscript to PLOS ONE. After careful consideration, we feel that it has merit but does not fully meet PLOS ONE's publication criteria as it currently stands. Therefore, we invite you to submit a revised version of the manuscript that addresses the points raised during the review process.

Our answer:

We thank you for the positive feedback. We have carefully considered the Reviewers' comments and suggestions and have revised our manuscript following each of these points.

Please submit your revised manuscript by Sep 30 2022 11:59PM. If you will need more time than this to complete your revisions, please reply to this message or contact the journal office at plosone@plos.org. When you're ready to submit your revision, log on to https://www.editorialmanager.com/pone/ and select the 'Submissions Needing Revision' folder to locate your manuscript file.

Our answer:

Thank you for this comment. We have submitted the revised manuscript on time.

Please include the following items when submitting your revised manuscript:

- **A rebuttal letter that responds to each point raised by the academic editor and reviewer(s). You should upload this letter as a separate file labeled 'Response to Reviewers'.**

- **A marked-up copy of your manuscript that highlights changes made to the original version. You should upload this as a separate file labeled 'Revised Manuscript with Track Changes'.**
- **An unmarked version of your revised paper without tracked changes. You should upload this as a separate file labeled 'Manuscript'.**

Our answer:

Thank you for this comment. We have included a rebuttal letter, a marked-up copy of the manuscript, and an unmarked version of the revised manuscript when submitting our revised manuscript.

If you would like to make changes to your financial disclosure, please include your updated statement in your cover letter. Guidelines for resubmitting your figure files are available below the reviewer comments at the end of this letter.

Our answer:

Thank you for this comment. We do not have any changes in our financial disclosure.

If applicable, we recommend that you deposit your laboratory protocols in protocols.io to enhance the reproducibility of your results. Protocols.io assigns your protocol its own identifier (DOI) so that it can be cited independently in the future. For instructions see: https://journals.plos.org/plosone/s/submission-guidelines#loc-laboratory-protocols. Additionally, PLOS ONE offers an option for publishing peer-reviewed Lab Protocol articles, which describe protocols hosted on protocols.io. Read more information on sharing protocols at https://plos.org/protocols?utm_medium=editorial-email&utm_source=authorletters&utm_campaign=protocols.

Our answer:

Thank you for this comment. Laboratory protocol does not apply to our study, but study protocol does. Our study protocol has included in the Methods section.

Journal Requirements:

Please review your reference list to ensure that it is complete and correct. If you have cited papers that have been retracted, please include the rationale for doing so in the manuscript text, or remove these references and replace them with relevant current references. Any changes to the reference list should be mentioned in the rebuttal letter that accompanies your revised manuscript. If you need to cite a retracted article, indicate the article's retracted status in the References list and also include a citation and full reference for the retraction notice.

Our answer:

Thank you for this comment. We have reviewed the reference list, which is complete and correct. There are not any retracted articles on the list.

RESPONSE TO REVIEWERS

6. Review Comments to the Author

Reviewer #1: The authors have greatly improved their manuscript. However, some issues remain to be addressed.

Our answer:

Thank the Reviewer so much for taking the time to leave the excellent reviews!

Major issues:

1) The authors name their analyses "multivariate", but I still do not identify the multivariate aspects. The authors analysed ICU and hospital mortality separately. Hence, they consider only one dependent variable at a time. Regarding the independent variables in the regression models, the authors build univariable and multivariable regression models, i.e. including one (univariable) or more than one (multivariable) independent variable. So, please clarify.

Our answer:

Thank you for this valuable comment that helped us understands further the terms of regression models. In each of our regression models, there was only one dependent variable (ICU or hospital mortality) at a time; thus, we have replaced the terms 'binary/univariate' or 'multivariate' with 'univariable' or 'multivariable' for these models throughout the manuscript.

2) One of my previous comments might have been not clear enough. As I asked about the way, according to which possible centre effects were accounted for in the regression models, this was not about the variable central hospital (yes/no), but about mixed effect logistic regression modelling with the centre as random effect. Did the authors consider this approach? If so, why did the authors decided not to apply it?

Our answer:

We are sorry for misunderstanding your comment in the previous revision of the manuscript. The concern of possible center effects, which you have raised, is valuable for our paper. To the best of our knowledge, the mixed-effects logistic regression model can be used to predict discrete outcome variables when observations are correlated. In our study, hospital mortality (dependent variable) was a primary outcome measured at two different time points, i.e., inside and outside the intensive care unit (ICU). Therefore, mixed effect logistic regression modeling with the center as a random effect is the most suitable for our analysis. However, our independent variables (e.g., qSOFA) were not measured at two corresponding time points. Therefore, this limitation prevented us from applying a mixed-effects logistic regression model. We have added this issue to the Limitation section as follows:

"Due to our independent variables (e.g., qSOFA) that might be associated with primary outcome (hospital mortality) only measured upon ICU admission, the mixed-effects logistic regression model could not be used to predict discrete outcome variables measured at two different times, i.e., inside and outside the ICU." (Lines 403-407)

3) The description of the modelling approach is still not completely clear.

a. Please clarify "candidate variables" in line 241/242.

Our answer:

Thank you for this comment. We have clarified “candidate variables” and have added this sentence to the Statistical analyses section as follows:

“Using a stepwise backward elimination method, we started with the full multivariable logistic regression model that included the selected variables.” (Lines 245-247)

b. Please clarify “some significant contribution to the outcome”. Which criteria apply? Please add all required information in the method and result section.

Our answer:

Thank you for this comment. We have clarified “some significant contribution to the outcome” and have added this sentence to the Statistical analyses section as follows:

“This method then deleted variables stepwise from the full model until all remaining variables were independently associated with the risk of death in the hospital.” (Lines 247-249)

c. Is there a link between step (b) and (c)? If so, please state. If not, some results are probably missing. Please clarify.

Our answer:

Thank you for this comment. There was a link between steps (b) and (c). Therefore, we have merged these steps into one and have stated them in the Statistical analyses section (Lines 239-249).

4) It is not clear, whether the authors additionally applied the originally proposed cut-off value for the qSOFA score to their own data. If this was done, please add this in the method and the results section. Otherwise, please perform this additional analysis and report it in the manuscript. Subsequently, this can be added to the discussion as well.

Our answer:

Thank you for this valuable comment that helped us improve the contents of this paper. We have performed additional analysis and reported it in the Method section (Lines 232-234), the Results section (Lines 311-316), and the Discussion section (Lines 382-388) of the manuscript.

Minor issues:

1) Line 82: Please add "in univariable and multivariable regression modelling" behind "factors associated with the hospital and ICU mortalities were assessed". Otherwise, the reader is surprised by the results (AOR) in the subsequent section.

Our answer:

Thank you for this comment. We have added "in univariable and multivariable logistic models" behind "factors associated with the hospital and ICU mortalities were assessed" (Lines 82-83).

2) Line 76/77: Please consider rewording. Suggestion: "[…] Vietnam on specified days […] representing the four different seasons of 2019."

Our answer:

Thank you for this comment. We have reworded this sentence (Line 76).

3) Line 90: OR should probably be AOR.

Our answer:

Thank you for this comment. This error is typing mistake. We have replaced 'OR' with 'AOR' (Line 91).

4) Line 128: "normal ward" instead of "ward", if this applies.

Our answer:

Thank you for this comment. We have replaced ‘ward’ with ‘normal ward’ (Line 129).

5) Line 156: One full stop too much. Please remove.

Our answer:

Thank you for this comment. We have removed a redundant punctuation mark (Line 157).

6) Line 160: Please clarify “representatives”. It becomes not clear, whether they are part of the local study team.

Our answer:

Thank you for this comment. Representatives are part of the local study team and the MOSAICS II study group. We have clarified ‘representatives’ as follows:

“Each ICU had one or two representatives who were part of the local study team and the MOSAICS II study group, as shown in eAppendix 2 of a previously published paper.[6]” (Lines 160-162)

7) Line 162: Please rephrase. Suggestion: “[…] on one of the four days […]”.

Our answer:

Thank you for this comment. We have rephrased this sentence. (Line 165)

8) Line 163: Please add 2019 behind the dates.

Our answer:

Thank you for this comment. We have added ‘2019’ behind the dates. (Line 166)

9) Line 171: Please clarify. Suggestion: "Data was entered by the representatives of the hospitals […]", if this applies. Otherwise, please clarify.

Our answer:

Thank you for this comment. We have clarified this sentence. (Lines 175-176)

10) Line 192: "or" instead of "and"?

Our answer:

Thank you for this comment. We have replaced 'and' with 'or'. (Line 197)

11) Line 216: Please write chi-2 with a 2 as superscript.

Our answer:

Thank you for this comment. We have rewritten this word. (Lines 220-221)

12) Please unify the number of decimal places. The number should be consistent for all median values and corresponding quartiles, for all percentages (always one decimal place, even for 0.0%) and so on. This applies for the text body as well as for tables and figures.

Our answer:

Thank you for pointing this out. We have unified the number of decimal places throughout the manuscript as well as the tables and figures; the number has been consistent for all median values and corresponding quartiles, for all percentages, and so on.

13) Please consider to write p_AUROC (_ should indicate, that AUROC should be a subscript) instead of p for p-values corresponding to AUCs. This would facilitate reading. This applies to main text as well as figure and table descriptions.

Our answer:

Thank you for this comment. We have written P_{AUROC} instead of p for p-values corresponding to AUCs throughout the manuscript, as well as figure and table descriptions.

14) Table 1:

a. Please indicate, if for a variable no p-value is provided (here: HIV infection), e.g. by -.

Our answer:

Thank you for this comment. We have added an abbreviation ‘NA’ that means not available for where no p-values provided.

b. Abbreviations n and no. are missing. Please add.

Our answer:

Thank you for this comment. We have added abbreviations ‘n’ and ‘no.’

15) Table 2: Abbreviations n and no. are missing. Please add.

Our answer:

Thank you for this comment. We have added abbreviations ‘n’ and ‘no.’

16) Table 3:

a. “Binary” is probably “univariable” and “multivariate” is probably “multivariable”. See comment above. Please adapt, if this applies. Otherwise, please clarify.

Our answer:

Thank you for this valuable comment that helped us understands further the terms of regression models. In each of our regression models, there was only one dependent variable

(ICU or hospital mortality) at a time; thus, we have replaced the terms ‘binary/univariate’ or ‘multivariate’ with ‘univariable’ or ‘multivariable’ for these models throughout the manuscript, as well as table descriptions.

b. Which kind of multivariable regression modelling was applied? Results of final model from backward elimination?

Our answer:

Thank you for this comment. We used the multivariable logistic regression model with the stepwise backward elimination method. Table 3 presents the results of the final multivariable logistic regression model from backward elimination. We have clarified this issue at the bottom of the table.

c. Please provide the reference to the complete regression results in the description. At least several univariable models are probably missing. If results from backward elimination are presented, then here should also be a link to the respective results in the supplement.

Our answer:

Thank you for this comment. We have provided the reference to the complete regression results in the description.

17) Table S1:

a. There seems to be some redundancy with Table 1. Please clarify. Is Table 1 just the short version for the main text body? Then, please refer in the description of Table 1 for additional information to Table S1.

Our answer:

Thank you for this comment. Yes, it is. Table 1 is just the short version of the main text body. We have referred to the description of Table 1 for additional information to Table S1.

b. Abbreviations n and no. are missing. Please add.

Our answer:

Thank you for this comment. We have added abbreviations ‘n’ and ‘no.’

18) Table S2:

a. There seems to be some redundancy with Table 2. Please check/clarify. Is Table 2 just the short version for the main text body? Then, please refer in in the description of Table 2 for additional information to Table S2.

Our answer:

Thank you for this comment. Yes, it is. Table 2 is just the short version of the main text body. We have referred to the description of Table 2 for additional information to Table S2.

b. “no. (%) and median (Q1-Q3), days”:

i. “and” should be “or”. Please check.

Our answer:

Thank you for this comment. We have replaced 'and' with 'or'.

ii. The comma should be removed. Please check.

Our answer:

Thank you for this comment. We have removed the comma.

c. Abbreviations n and no. are missing. Please add.

Our answer:

Thank you for this comment. We have added abbreviations ‘n’ and ‘no.’

19) Table S3: Abbreviations n and no. are missing. Please add.

Our answer:

Thank you for this comment. We have added abbreviations ‘n’ and ‘no.’

20) Table S4:

a. “no. (%) and median (Q1-Q3) days”: “and” should be “or”.

Our answer:

Thank you for this comment. We have replaced 'and' with 'or'.

b. Abbreviations n and no. are missing. Please add.

Our answer:

Thank you for this comment. We have added abbreviations ‘n’ and ‘no.’

21) Table S5: Please see for “bivariate regression analyses” comments above.

Our answer:

Thank you for this valuable comment that helped us understands further the terms of regression models. In each of our regression models, there was only one dependent variable (ICU or hospital mortality) at a time; thus, we have replaced the terms ‘binary/univariate’ or ‘multivariate’ with ‘univariable’ or ‘multivariable’ for these models throughout the manuscript, as well as table descriptions.

22) Table S6: Please see for “bivariate regression analyses” comments above.

Our answer:

Thank you for this valuable comment that helped us understands further the terms of regression models. In each of our regression models, there was only one dependent variable (ICU or hospital mortality) at a time; thus, we have replaced the terms ‘binary/univariate’ or ‘multivariate’ with ‘univariable’ or ‘multivariable’ for these models throughout the manuscript, as well as table descriptions.

23) Table S7:

a. Please see for “multivariate logistic regression analyses” comments above.

Our answer:

Thank you for this valuable comment that helped us understands further the terms of regression models. In each of our regression models, there was only one dependent variable (ICU or hospital mortality) at a time; thus, we have replaced the terms ‘binary/univariate’ or ‘multivariate’ with ‘univariable’ or ‘multivariable’ for these models throughout the manuscript, as well as table descriptions.

b. Please add the number of patients included in the models.

Our answer:

Thank you for this comment. We have added the number of patients included in the models.

c. “Unit” is still not clear. Was modelling performed on individual patients’ data or already on aggregated data? If the analysis was done on individual level data, it is unclear, how a patient, for example, can only be in parts older than 65 years of age. A patient is either below 65 years or above, but cannot be a mixture of both. These questions are also related to the comment above on a more detailed method description.

Our answer:

Thank you for this comment. We have replaced 'Unit' with 'Number of patients'.

24) Table S8: Please see comments for Table S7.

Our answer:

Thank you for this comment. We have addressed the concerns you raised.

25) Figure 1: Please introduce the word “abbreviations:” in front of the list of abbreviation (as in Figure S1).

Our answer:

Thank you for this comment. We have addressed the concerns you raised.

26) Figure S2: Please see comment for Figure 1.

Our answer:

Thank you for this comment. We have addressed the concerns you raised.

27) Figure S3: Please see comment for Figure 1.

Our answer:

Thank you for this comment. We have addressed the concerns you raised.

28) Figure S4: Please see comment for Figure 1.

Our answer:

Thank you for this comment. We have addressed the concerns you raised.

Reviewer #2: (No Response)

We thank the Reviewer for the valuable comments and suggestions that helped us improve the contents of this paper.

Reviewer #4: Reviewer comments

Manuscript Number: PONE-D-21-40686_R1

Title "Predictive validity of the quick Sequential Organ Failure Assessment (qSOFA) score for the mortality in patients with sepsis in Vietnamese intensive care units".

Thank you for providing me the opportunity to review this revised manuscript that raises important issues about predictive validity of the quick Sequential Organ Failure Assessment (qSOFA) score for the mortality in patients with sepsis in intensive care units in one of the developing countries.

It seems that all corrections were done.

Our answer:

We thank the Reviewer for the valuable comments and suggestions that helped us improve the contents of this paper.

Thank the Editors and Reviewers so much for taking the time to leave their excellent reviews!

Sincerely yours,

Chinh Quoc Luong, MD., PhD.
Center for Emergency Medicine,
Bach Mai Hospital,
No. 78, Giai Phong, Phuong Mai ward, Dong Da district, Hanoi 100000, Vietnam
Email: luongquocchinh@gmail.com
On behalf of all authors
