# Supplementary material for: Predictive validity of the quick Sequential Organ Failure Assessment (qSOFA) score for the mortality in patients with sepsis in Vietnamese intensive care units
Source: PLoS One. 2022 Oct 14;17(10):e0275739. doi: 10.1371/journal.pone.0275739 (PMC9565713; doi:10.1371/journal.pone.0275739)
Supplement: Attachment — Submitted filename: Reviewer comments PONE-D-21-40686.docx [file pone.0275739.s004.docx]

Reviewer comments

Manuscript Number: PONE-D-21-40686

Title "Predictive validity of the quick Sequential Organ Failure Assessment (qSOFA) score for the mortality in patients with sepsis in Vietnamese intensive care units".

**Generally speaking:**
Thank you for providing me the opportunity to review this manuscript that raises important and interesting issues about predictive validity of the quick Sequential Organ Failure Assessment (qSOFA) score for the mortality in patients with sepsis in intensive care units in one of the developing countries.

**Comment 1:**

1. *ABSTRACT:*
a) No need for details of methodology in the abstract. Only main points. It should not include the type of statistical analysis.
b) Methodology has to mention the elements included as measuring parameter but without details.
c) Abbreviations as APACHE, MOSAICS, and SIRS must be defined upon first appearance in the keywords.
d) It is preferable that the keywords should be 5-6 words only.

**Comment 2:**
*2. INTRODUCTION:*

a) Global/ Regional/ Vietnam prevalence of the mortality of ICU patients with sepsis should be mentioned.
b) Factors associated with mortality in patients with sepsis upon Vietnamese intensive care unit admission should be clearly stated.
c) Explaining why this topic was chosen for analysis in this article is not well written. The benefits of conducting the study to the community should be explained.

**Comment 3:**
*3. METHODS:*

a) Were the samples normal or not?
b) Tests of significant for each type of variables should be mentioned.
c) In lines 158 and 159, why predominantly neurosurgical, coronary, and cardiothoracic ICUs were excluding?
d) The basis of sample size calculation should be clearly stated to know the confidence level and the margin of error.

**Comment 4:**
*4. RESULTS:*

a) No need to conduct bivariate logistic regression because it doesn’t consider the confounding variables.
b) What was the criteria to pass your variables from bivariate to multivariate logistic regression?

**Comment 5:**

*5. DISCUSSION:*

a) It is advisable to explain the study objective at the beginning of the discussion.
b) Compare the findings of the study with other findings and state the reasons for the strengths and weaknesses in each section. The manuscript could be greatly strengthened if the authors could provide highlight on the **main** **and significant** **factors associated with hospital and ICU mortalities** **in patients with sepsis** in other developing and developed countries with similar context.
c) The manuscript could also be greatly strengthened if the authors could provide highlight on the **main** **and significant life-sustaining treatments during ICU stay and outcomes of ICU patients with sepsis according to hospital survivability** in other developing and developed countries with similar context.
d) In brief, discuss by using the scientific reasoning the differences in accuracy of the qSOFA score to predict the risk of hospital and ICU deaths from sepsis.
e) In lines 305 and 306, the sentence “Our 304 figures for the hospital and ICU mortality rates are in line with the figures reported in the 305 worldwide Intensive Care over Nations (ICON) study (19.3% [26/439] to 47.2% [17/141], and 11.9% [16/439] to 39.5 [15/141], respectively)” was not clearly stated.
f) Abbreviations as HIC must be defined upon first appearance in the discussion.

**Comment 6:**

*6. CONCLUSION:*

Please write suggestions for improvement.

**Comment 7:**

*7. STRENGTHS AND LIMITATIONS:*

Strengths and limitations of the study should be analyzed in separate paragraphs.
