# Supplementary material for: Predictive validity of the quick Sequential Organ Failure Assessment (qSOFA) score for the mortality in patients with sepsis in Vietnamese intensive care units
Source: PLoS One. 2022 Oct 14;17(10):e0275739. doi: 10.1371/journal.pone.0275739 (PMC9565713; doi:10.1371/journal.pone.0275739)
Supplement: Attachment — Submitted filename: Reviewer comments PONE-D-21-40686_R1.docx [file pone.0275739.s006.docx]

Reviewer comments

Manuscript Number: PONE-D-21-40686_R1

Title "Predictive validity of the quick Sequential Organ Failure Assessment (qSOFA) score for the mortality in patients with sepsis in Vietnamese intensive care units".

Thank you for providing me the opportunity to review this revised manuscript that raises important issues about predictive validity of the quick Sequential Organ Failure Assessment (qSOFA) score for the mortality in patients with sepsis in intensive care units in one of the developing countries.

It seems that all corrections were done.
